# Bimodal mortality dynamics for uveal melanoma: a cue for metastasis development traits?

**DOI:** 10.1186/1471-2407-14-392

**Published:** 2014-06-02

**Authors:** Romano Demicheli, Marco Fornili, Elia Biganzoli

**Affiliations:** 1 Scientific Directorate, Fondazione IRCCS Istituto Nazionale Tumori di Milano, Milano 20133, Italy; 2 Medical Statistics and Biometry, Università di Milano, Milano 20133, Italy; 3 Medical Statistics and Biometry, Università di Milano e Fondazione IRCCS Istituto Nazionale Tumori di Milano, Milano 20133, Italy

## Abstract

**Background:**

The study estimates mortality dynamics (event-specific hazard rates over a follow-up time interval) for uveal melanoma.

**Methods:**

Three thousands six hundred seventy two patients undergoing radical or conservative treatment for unilateral uveal melanoma, whose yearly follow-up data were reported in three published datasets, were analysed. Mortality dynamics was studied by estimating with the life-table method the discrete hazard rate for death. Smoothed curves were obtained by a Kernel-like smoothing procedure and a piecewise exponential regression model. The ratio deaths/patients at risk per year was the main outcome measure.

**Results:**

The three explored hazard rate curves display a common bimodal pattern, with a sudden increase peaking at about three years, followed by reduction until the sixth-seventh year and a second surge peaking at about nine years after treatment.

**Conclusions:**

The bimodal pattern of mortality indicates that uveal melanoma metastatic development cannot be explained by a continuous growth model. Similar metastasis dynamics have been reported for other tumours, including early breast cancer, for which it supported a paradigm shift to an interrupted growth model, the implications of which are episodes of ‘tumour dormancy’. We propose that the concepts of tumour homeostasis, tumour dormancy and enhancement of metastasis growth related to primary tumour removal, convincingly explaining the clinical behaviour of breast cancer, may be used for uveal melanoma as well. To confirm this proposition, a careful analysis of uveal melanoma metastasis dynamics is strongly warranted.

## Background

Thirty four years ago Lorenz E Zimmerman, Ian W McLean, and Walter D Foster published the landmark article “Does enucleation of the eye containing a malignant melanoma prevent or accelerate the dissemination of tumour cells?” [1]. In that article the authors put forward a hypothesis casting doubts on the benefit of enucleation that was the prevailing management of choroidal melanoma at that time. Their work stimulated the development of alternative therapies including various forms of radiotherapy and tumour resection, resulting in successive wide diffusion of “conservative” treatments [2]. Their hypothesis was primarily based on the mortality pattern, estimated from an historical series, which apparently peaked 2–3 years after enucleation and stabilised by the seventh year of follow-up. The clinical, epidemiological, statistical, and experimental evidence developed over the elapsed 34 years since the publication of the hypothesis confirms the phenomenon of transient rise in post-therapeutic mortality. Although most ophthalmologists do not presently attribute this fact to the procedure of enucleation, and believe that it can be explained on the basis of early metastasis, questions implied by the Zimmerman-McLean-Foster hypothesis are still valid [3].

The sudden emergence of metastatic disease in the early years following primary tumour removal is not limited to uveal melanoma. Similar recurrence patterns have been reported for early breast cancer [4], non-small-cell lung cancer (NSCLC) [5] and head and neck cancer [6], for which an additional later recurrence peak is also detectable. For breast cancer, this result confirmed the previous evidence that continuous tumour growth is irreconcilable with clinical findings [7] and supported a paradigm shift to an interrupted growth model, the implications of which are episodes of ‘tumour dormancy’. According to the new paradigm, primary tumour removal, usually considered as intrinsically beneficial, can perturb metastatic homeostasis and, for some patients, results in the acceleration of metastatic development [8]. The occurrence of a second mortality peak for choroidal melanoma was suggested from RBS Packard [9] more than 30 years ago. To our knowledge, however, the issue was not further addressed by other investigators.

As data of uveal melanoma annual mortality are available in a few published reports, we performed an analysis of the hazard rate for mortality with the same technique adopted to estimate breast cancer event dynamics (event-specific hazard rates over a follow-up time interval). Such an analysis, aimed at exploring analogies between the clinical courses of these different neoplasias possibly relying on similar metastasis behaviours, is the object of present report.

## Methods

**Figure 1:**
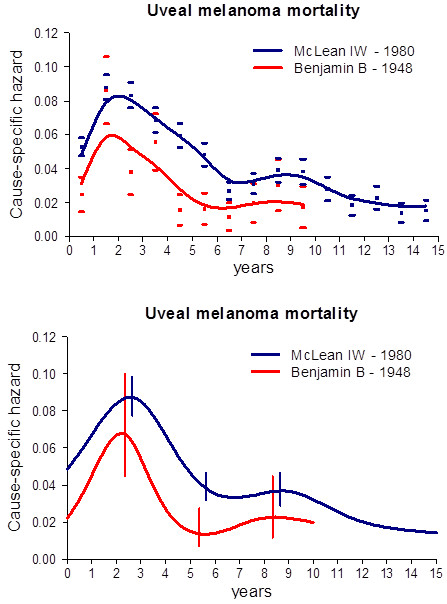
**Cause-specific hazard rate estimates for mortality in patients undergoing eye enucleation for uveal melanoma.** Blue line: data on 2105 patients from the McLean series. Red line: data on 250 patients from Benjamin series. Time-origin is at surgery. **(Upper panel)** Smoothed curves are obtained by a Kernel-like smoothing procedure. Standard deviation estimates for single points are also reported. **(Lower panel)** Hazard rates obtained by the piecewise exponential regression model. Vertical lines represent 95% point-wise confidence intervals.

A first set of data on the follow-up of patients undergoing enucleation for uveal melanoma were directly drawn from Table 1 of the report from Mc Lean IW et al [10]. The table encloses the annual number of patients either died from melanoma metastasis or censored, among 2105 patients recorded in the Registry of Ophthalmic Pathology of the Washington Hospital Centre, mostly between 1940 and 1960. A second dataset, from the Royal Ophthalmic Hospital and Royal Eye Hospital of London on 250 patients treated for uveal melanoma between 1925 and 1939, was found in Table four of the paper from Benjamin B et al. [11]. The table encloses annual numbers of deaths from melanoma metastasis and average numbers of patients at risk. The third dataset originates from the randomized multicentre clinical trial of brachytherapy versus enucleation conducted by the Collaborative Ocular Melanoma Study (COMS) Group on 1317 patients with medium-sized choroidal melanoma, enrolled from 1986 to 2003, and was obtained from Figure 1 of the COMS report N° 28 [12]. The dataset provides the number of deaths with histopathologically confirmed melanoma metastasis and the number of censored events. Data from the COMS clinical trial of enucleation versus pre-enucleation radiation therapy for large tumours was not considered suitable for the analysis due to the insufficient follow-up of the available dataset (COMS report N° 24 [13]). Patient and tumour characteristics are available in the reported papers.

**Table 1:** Patient characteristics of the studied uveal melanoma series

	**Mc Lean series**	**Benjamin series**	**COMS series**
Number of patients	2055	250	1317
Sex (Males/females)	Not reported	116/134	665/652
Mean age (Years)	55	54	60
Tumour size^1^ (%)			
Small	27		35
Medium	43	Not reported	57
Large	30		8
Cell type (%)			
Spindle cell	45	64	Not reported
Mixed cell^2^	55	36	

1–Small: ≤ 10 mm; medium: 11–15 mm; large: >15 mm.

2–Mixed cell: includes the mixed, fascicular and epithelioid type of the Callender classification.

Mortality dynamics was studied by estimating with the life-table method the discrete hazard rate for death, *i.e.*, the conditional probability of dying in a time interval, given that the patient was alive at the beginning of the interval. A discretization of the time axis in one year units was applied. Therefore, all hazard rate levels were measured as ‘events/patients at risk per year’. Since the hazard rate estimates display some instability due to random variation, a Kernel-like smoothing procedure was adopted and the smoothed curve was graphically represented to make it easier for the reader to perceive the underlying pattern [14]. This method, which is akin to a moving average, estimates the events/exposure rate at each time point by a local weighted mean of the observations.

In addition to the Kernel smoothing approach with discrete hazards, a flexible parametric approach based on the piecewise exponential model was also adopted in order to obtain smoothed hazard estimates [15]. Natural cubic splines, i.e., with linearity constraints on the tails, were used with knots placed equidistantly within the respective year ranges. The number of knots, corresponding to a number of basis functions between 3 and 8, was chosen by minimizing the Akaike Information Criterion (AIC). AIC estimates the relative distance between the fitted and the true model, and increases when unnecessary flexibility is added to the model. This criterion is suitable for model selection even when fitted models are not nested, as is the case when splines knots locations change according to their number [16]. The 95% point-wise confidence intervals were based on the log transformation. For a comparison with a regularized (e.g., smoothing) regression approach, P-splines [17] were also used, where a maximum number of knots is chosen from the beginning, and, to avoid overfitting, a term penalizing the roughness of the estimator is introduced. The trade-off between fit to observed data and curve smoothness is regulated by a penalty parameter.

## Results

A few patient and tumour characteristics are reported in Table 1. The hazard rate pattern for uveal melanoma mortality for the McLean and Benjamin series is reported in Figure 1. The two explored hazard rate curves display a common bimodal pattern, with a sudden increase peaking at about three years, followed by less rapid reduction until the sixth-seventh year and a second surge peaking at about nine years after enucleation. This pattern is well detectable in the Benjamin series, in spite of the less extended follow-up. Of note, this older series displays a lower hazard level in comparison with the more recent McLean series, yet maintaining the same overall pattern.

**Figure 2:**
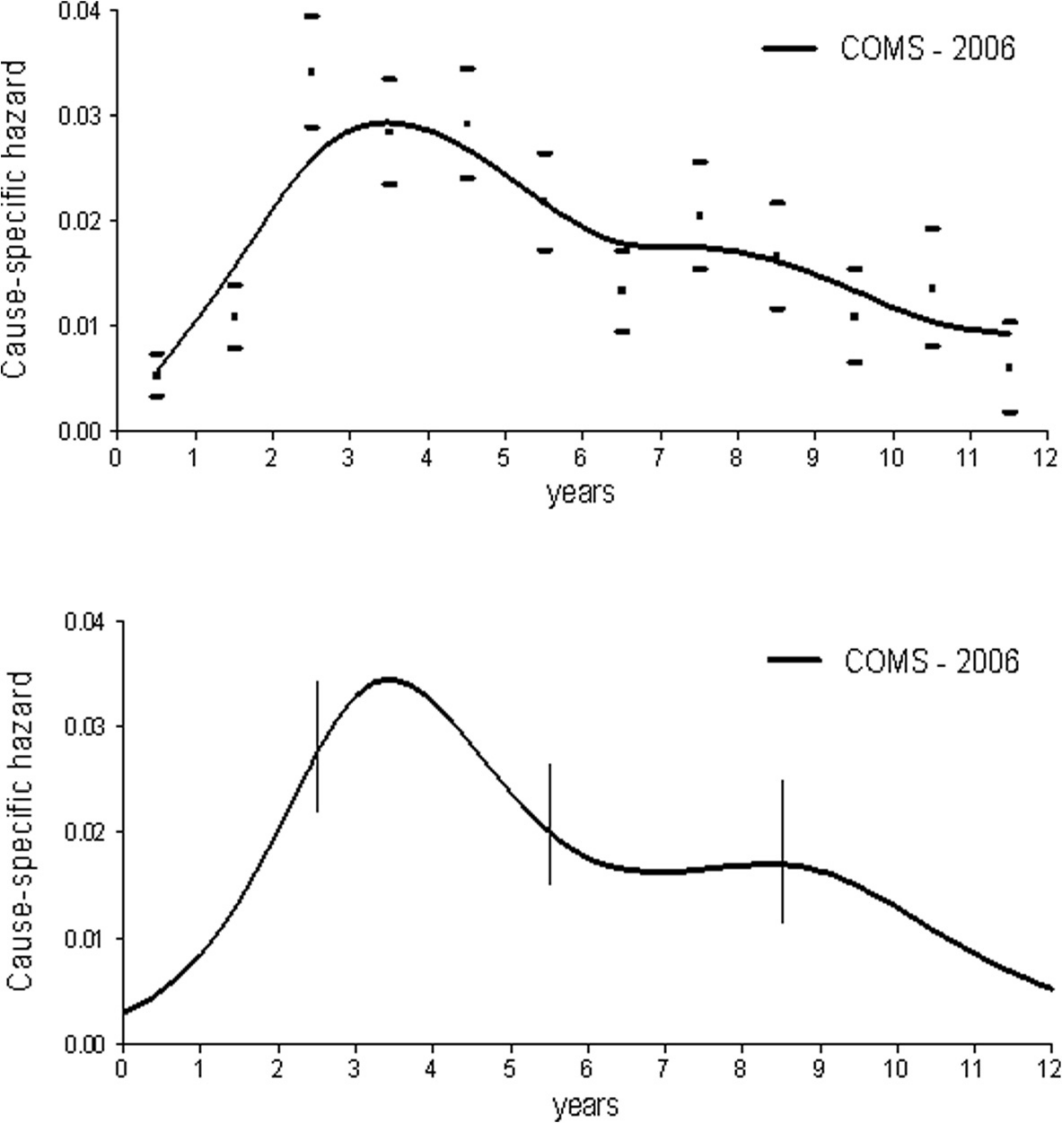
**Cause-specific hazard rate estimates for mortality in 1317 patients from the COMS series, undergoing either I**^**125 **^**brachytherapy or enucleation for unilateral choroidal melanoma.** Time-origin is at surgery. **(Upper panel)** Smoothed curves are obtained by a Kernel-like smoothing procedure. Standard deviation estimates for single points are also reported. **(Lower panel)** Hazard rates obtained by the piecewise exponential regression model. Vertical lines represent 95% point-wise confidence intervals.

The COMS randomized study confirmed that there is no survival difference between patients whose tumours are treated with radical treatment and those treated with conservative treatment. Consequently, the two series were merged and jointly analysed, after confirmation that the recurrence dynamics for patients undergoing brachytherapy is similar to the corresponding pattern for enucleated patients (data not shown) (Figure 2). The recurrence dynamics for these patients once again displays the bimodal pattern, although with minor differences in the peak timing when compared with the older series, an occurrence that may be compatible with different data collection accuracies.

## Discussion

**Figure 3:**
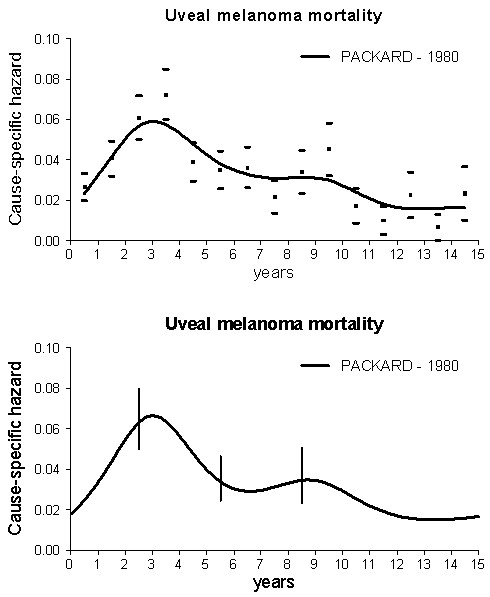
**Cause-specific hazard rate estimates for mortality in 578 patients treated at the Moorfields Eye Hospital between 1949 and 1972 for Choroid melanoma.** Time-origin is at surgery. **(Upper panel)** Smoothed curves are obtained by a Kernel-like smoothing procedure. Standard deviation estimates for single points are also reported. **(Lower panel)** Hazard rates obtained by the piecewise exponential regression model. Vertical lines represent 95% point-wise confidence intervals.

Our analysis provides evidence that the mortality dynamics for patients undergoing enucleation for uveal melanoma displays a bimodal pattern (Figure 1). Moreover, similar mortality dynamics was detected for patients with choroidal melanoma treated with either surgery or radiation therapy (Figure 2). It should be noted that the characteristics of the two series display dissimilarities in the frequency of a few prognostic parameters (Table 1), although all examined patients shared the common basic trait of bearing a tumour that could be dealt with enucleation. The occurrence that a bimodal pattern was detected in datasets from different institutions, regarding patients with different characteristics treated during different time spans and with different modalities strongly supports the validity of the result that emerges in spite of possible confounding factors. Of note, moreover, if the mortality data reported by RBS Packard [9] are analysed with the same technique adopted by us, the same bimodal mortality pattern evidently emerges (Figure 3).

Similar pattern was formerly detected for early breast cancer [4] and, more recently, for NSCLC [5] and head and neck cancer [6]. In particular, this finding raises the possibility that major analogies may exist between uveal melanoma and early breast cancer metastasis biology, although the latter was inferred from recurrence dynamics rather than from mortality. It is very reasonable, however, to assume that uveal melanoma recurrence dynamics have a bimodal pattern as well, although with earlier peak timing in comparison with mortality. Indeed about 90% of recurring uveal melanoma patients display metastatic liver involvement with median survival time of less than 6 months from metastasis detection, largely due to the lack of effective treatments for metastatic disease [18]. The assumption is also strengthened by the finding that breast cancer mortality dynamics still maintains bimodal pattern, in spite of the fact that survival following recurrence is much more prolonged and displays high inter-patient variability [19]. Uveal melanoma and early breast cancer, moreover, display a few other remarkable clinical similarities, supporting analogy in metastasis biology, at least after surgical treatment: (i) uncommon observation of metastases at diagnosis; (ii) complete primary tumour removal; (iii) early recurrence after surgery in a substantial number of patients; (iv) prolonged risk of recurrence, with late metastasis emergence even after decades. Additionally, the occurrence that in patients with choroidal melanoma similar mortality dynamics is detectable after surgery or radiotherapy suggests that in ocular melanoma similar metastatic development biology may occur following the two different primary tumour treatments.

On the basis of such analogies, we may hypothesize that the concepts of tumour homeostasis, tumour dormancy and surgery related enhancement of metastasis growth, convincingly explaining the clinical behaviour of breast cancer [8], may be used for uveal melanoma as well. According to this biology, following tumour cell shedding from the primary, the development of metastases includes sequential passage through a few phases: (a) single mostly non-dividing tumour cells (cellular dormancy), (b) non-angiogenic micro-metastases (and angiogenic ones in the presence of anti-angiogenic factors) that cannot grow more than the size of avascular foci (micrometastatic dormancy) and (c) vascularised growing metastases doomed to reach the clinical level. This orderly process is controlled by the primary tumour that can exert restraints on the transition between dormancy phases by not yet well elucidated mechanisms, thus retarding or inhibiting metastasis development. Surgical removal (and brachytherapy for choroidal melanoma) of primary tumour may, therefore, disrupt such a tumour homeostasis [20], with sudden synchronization and acceleration of the metastatic process, at least for some patients, and originate the first mortality peak. Residual single dormant cells and avascular micrometastases, the amount of which cannot increase further due primary tumour removal, will follow an unperturbed course resulting in the late mortality peak. Details of the process supporting the acceleration of metastasis are not yet well understood. Both the treatment trauma *per se* (activation of the healing programme) and the primary tumour removal (disruption of homeostatic equilibrium) are strongly suspected to be relevant. Angiogenesis induction by shifted balance between enhancing and inhibiting factors may be an important, but not exclusive, molecular mechanism for dormancy interruption, while, on the basis of present results, current treatment modalities of primary tumour apparently seem to play a minor role.

A comment is needed on the question of why Zimmerman and colleagues did not report a bimodal pattern in their analysis [1]. It should be noted that they obtained a least-square fit to the observed data by a mathematical model assuming a series of *a priori*, among which, of note, continuous tumour growth. If one looks at the experimental points in their Figure 3, the occurrence of a second surge of annual death rate at the 9th year is quite evident. A different more flexible smoothing approach would have probably called their attention on the late structure of the mortality pattern. Similar considerations may be put forward for the mortality analysis carried out in another study on a dataset partially overlapped to the McLean series [21].

Zimmerman, McLean, and Foster attributed the phenomenon they identified to the modalities of the surgical manoeuvre and hoped that, by paying attention to the details of enucleation and by irradiating the eye before this procedure, one might improve the prognosis of patients with uveal melanoma. Yet, all subsequent research efforts failed to provide evidence of prognosis improvement by technique refinements of the primary treatment. According to our proposed paradigm, this failure is perfectly explainable, since the reason for the early rise of recurrence and mortality after treatment of the primary is related to the treatment itself, not to technical details by which it is carried out.

For early breast cancer, peak timing is rather constant for all studied patient subsets while risk levels are greatly influenced by tumour and host traits [8]. The double peaked risk profile of a given patient is seemingly determined by a unique mix of tumour-host factors controlling the ability of tumour cells to progress throughout successive dormant states. Uveal melanoma might display similar behaviour: some factors may result from the tumour originating cell (e.g., gene profile or chromosomal anomalies such as chromosome 3 deletion), or by tumour traits (e.g., location or size) while others may result from host traits (e.g., sex, age) [22]. This occurrence is somehow anticipated by the report from RBS Packard [9] where bimodality is detectable in all analysed subsets (by duration of history, position and size of the tumour, cell type and scleral extension), in spite of the relatively naïve analysis. Further studies on this subject are needed. In particular, the fact that microarray-based gene expression profiling is able of discriminating two molecular prognostic classes (aggressive vs less aggressive) [23] should be carefully examined. This point recalls a similar question involving breast cancer prognosis, which may be related to oestrogen receptor (ER) status (ER positive vs ER negative), Her2 expression (Her2 positive vs Her2 negative) or to a combined classifier (triple negative vs others). Since the bimodal recurrence dynamics for breast cancer patients was detected in all subsets [24,25], without supporting the evidence for a “two population” hypothesis, in the absence of a (required) direct analysis of data we are inclined to think that for uveal melanoma also these prognostic classes may impact on peak height, not on peak position.

## Conclusions

Our analysis reveals that the mortality dynamics for patients undergoing enucleation or brachytherapy for uveal melanoma displays a bimodal pattern. Although the occurrence of a second mortality peak was suggested more than 30 years ago, the issue was not further addressed by other investigators and no reasonable explanation was put forward. Our investigation, which confirms that this pattern is detectable in datasets from different institutions and regarding patients treated during different time spans and with different modalities, strongly supports the validity of the result.

We suggest that the concepts of tumour homeostasis, tumour dormancy and surgery related enhancement of metastasis growth, convincingly explaining the clinical behaviour of breast cancer, may be used for uveal melanoma as well.

Results of the study have potential implications on treatment approaches and surveillance, which might indeed benefit from time-related recurrence rate modifications. We acknowledge, however, that confirmation of the proposed new paradigm for uveal melanoma needs careful analyses of the recurrence dynamics by patient and tumour traits. Such investigations are strongly warranted.

## Abbreviations

NSCLC: Non-small-cell lung cancer; COMS: Collaborative ocular melanoma study; AIC: Akaike information criterion.

## Competing interests

The authors declare that they have no competing interests.

## Authors’ contributions

RD conceived the study, contributed to data collection, result’s discussion and helped to draft the manuscript; MF contributed to data collection, performed statistical analysis and helped to draft the manuscript; EB contributed to statistical analysis, result’s discussion and helped to draft the manuscript. All authors read and approved the final manuscript.

## Pre-publication history

The pre-publication history for this paper can be accessed here:

http://www.biomedcentral.com/1471-2407/14/392/prepub
